# Therapeutic efficacy of α-radioimmunotherapy with different activity levels of the ^213^Bi-labeled monoclonal antibody MX35 in an ovarian cancer model

**DOI:** 10.1186/s13550-017-0283-2

**Published:** 2017-04-24

**Authors:** Anna Gustafsson-Lutz, Tom Bäck, Emma Aneheim, Ragnar Hultborn, Stig Palm, Lars Jacobsson, Alfred Morgenstern, Frank Bruchertseifer, Per Albertsson, Sture Lindegren

**Affiliations:** 1 Department of Radiation Physics, Institute of Clinical Sciences, The Sahlgrenska Academy, University of Gothenburg, Gula Stråket 2B, 413 45 Gothenburg, Sweden; 2 Department of Oncology, Institute of Clinical Sciences, The Sahlgrenska Academy, University of Gothenburg, Blå Stråket 2, 413 45 Gothenburg, Sweden; 3 European Commission, Joint Research Centre, Institute for Transuranium Elements, Hermann-von-Helmholtz-Platz 1, 76344 Eggenstein-Leopoldshafen, Germany

**Keywords:** Radioimmunotherapy, Dosimetry, High LET radiation, Monoclonal antibodies (mAb), Radiopharmaceuticals

## Abstract

**Background:**

The aim of this study was to compare the therapeutic efficacy of two different activity levels of the ^213^Bi-labeled monoclonal antibody MX35 in an ovarian cancer model. Sixty female BALB/c (nu/nu) mice were inoculated intraperitoneally with human ovarian cancer cells (OVCAR-3). Two weeks later, 40 mice were injected intraperitoneal (i.p.) with 1 ml of ^213^Bi-MX35, 3 MBq/mL (*n* = 20), or 9 MBq/mL (*n* = 20). An additional 20 mice received unlabeled MX35. Incidence of tumors and ascites was investigated 8 weeks after therapy. Body weight and white blood cell counts were monitored after treatment for possible signs of toxicity.

**Results:**

The tumor-free fraction of the animals treated with 3 MBq/mL of ^213^Bi-MX35 was 0.55, whereas that of animals treated with 9 MBq/mL of ^213^Bi-MX35 was 0.78. The control group treated with unlabeled MX35 had a tumor-free fraction of 0.15. No significant reduction in white blood cell counts or weight loss was observed.

**Conclusions:**

Tumor growth after i.p. treatment with ^213^Bi-MX35 was significantly reduced compared to treatment with unlabeled MX35. Treatment with 9 MBq/mL of ^213^Bi-MX35 resulted in higher tumor-free fraction compared with 3 MBq/mL of ^213^Bi-MX35, but this difference was not statistically significant. No signs of toxicity were observed in the treated animals.

## Background

The current therapy for ovarian cancer includes surgery and systemic chemotherapy, and although this approach is usually initially successful, a majority of the patients suffer from recurrence. Tumors occur mainly within the peritoneal cavity, which at late stages leads to ascites production, bowel obstruction, and eventual death. Only 45% have a 5-year relative chance of surviving ovarian cancer [1]. Consequently, additional therapy methods are urgently needed to increase the survival rate.

In an effort to meet this need, our research group has focused on intraperitoneal adjuvant alpha-particle radioimmunotherapy (α-RIT) with ^211^At and ^213^Bi [2–9]. Successful preclinical studies with ^211^At have already led to a clinical phase I study [10, 11], and additional clinical trials are planned. While most of our efforts have been spent on development and evaluation of ^211^At radiopharmaceuticals, model calculations [12] show that ^213^Bi might be preferential for intraperitoneal (i.p.) radioimmunotherapy (RIT) of disseminated ovarian cancer. The short half-life in combination with relatively long retention time in the peritoneal cavity of the patient allows administration of higher activities, and the resulting irradiation from unbound ^213^Bi-antibodies in the surrounding i.p. fluid could substantially contribute to a high absorbed dose to microtumors.

We have previously compared, head-to-head, the therapeutic efficacy and biodistribution of ^213^Bi- and ^211^At-labeled monoclonal antibodies (mAbs) in an ovarian xenograft cancer mouse model [13]. In that study, the administered activity concentrations of ^213^Bi and ^211^At were chosen to translate to approximately equal absorbed doses to the peritoneal lining in a corresponding patient situation, i.e., 7–8 Gy. The therapeutic results between the ^213^Bi-RIT and ^211^At-RIT could not be significantly distinguished. We have, however, also previously shown that the peritoneal lining in mice may tolerate absorbed doses as high as 30–50 Gy [13], implying the possibility of administering higher activity concentration levels in humans.

The current study was designed to evaluate if higher administered activity concentrations of ^213^Bi-mAb could improve the therapeutic efficacy of ^213^Bi-RIT in an ovarian cancer mouse model. A second aim was to evaluate if such improvement could be explained by the calculated absorbed dose to microtumors when including both cell-bound and free ^213^Bi-mAb irradiation.

The therapeutic efficacy was analyzed by rigorous examination of tumor incidence in the mice 8 weeks after treatment, to evaluate the tumor-free fraction for the different activity concentration levels. The highest activity concentration level administered in this study was calculated to be a safe absorbed dose to the bone marrow in mice.

## Methods

### Radionuclide production

The ^213^Bi was produced by a ^225^Ac/^213^Bi generator as described previously [14, 15] and was eluted from the generator according to the standard protocol provided by Institute for Transuranium Elements (ITU) in Karlsruhe, Germany. In short, 600 μL of a 0.1 M HCl/0.1 M NaI solution was pumped through the generator column to elute the ^213^Bi. Subsequently, the ^213^Bi solution was pH-adjusted by addition of 4 M sodium acetate and a 20% L-ascorbic acid solution (the ascorbic acid additionally protects the antibody conjugate from radiolysis) [16].

### Antibody

MX35, a murine IgG_1_ mAb, was used in the experiments. The MX35 mAb was developed at the Sloan-Kettering Cancer Center (New York, NY, USA) [17, 18] and was produced from hybridoma cells obtained from the Ludwig Institute for Cancer Research, Zürich, Switzerland. It recognizes the transport protein NaPi2b, which is homogeneously expressed in approximately 90% of human epithelial ovarian cancer cells and to only a small degree in normal tissues [19], making it suitable for epithelial ovarian cancer therapy.

### Cell line

The ovarian carcinoma cell line OVCAR-3 (American Type Culture Collection, Rocksville, MD, USA), was used in all in vitro and in vivo experiments. The cells were cultured in T-75 culture flasks in RPMI 1640 medium supplemented with 10% fetal calf serum, 1% L-glutamine, and 1% penicillin-streptomycin and were grown in a humidified atmosphere of 95% air/5% CO_2_ at 37 °C.

### Animals

Female immunodeficient BALB/c (nu/nu) mice (Charles River Laboratories International, Freiburg, Germany), 5–6 weeks of age, were used in the in vivo experiments. The housing of the animals was as previously described [13]. The animal study was performed according to the guidelines from the ethical committee and the legislations for animal research in Sweden and with approval from the Committee on the Ethics of Animal Experiments of the University of Gothenburg, Sweden.

### Antibody conjugation

The antibody-chelator conjugation was performed essentially as described previously [13], at room temperature (RT), overnight, in 0.2 M carbonate buffer, pH ~8.5. The chelator [(R)-2-amino-3-(4-isothiocyanatophenyl)propyl]-*trans*-(S,S)-cyclohexane-1,2-diamine-pentaacetic acid (CHX-A′′-DTPA) in dimethyl sulfoxide (0.018 M) was added to 2–4 mg/mL of mAb in 15 × molar excess. Subsequently, the buffer was changed to 0.1 M citrate, pH 5.5, using a NAP-5 column (GE Healthcare, Buckinghamshire, UK). For long-term storage, the buffer of the antibody conjugate was changed to phosphate buffered saline (PBS). The equipment was essentially kept metal-free, and the buffers included were made metal-free by incubation with Chelex 100 resin (Bio-Rad Laboratories, Hercules, CA, USA) as described previously [13]. The number of available chelators attached after labeling was estimated to be two per antibody according to evaluation by an arsenazo III spectrophotometric assay [20].

### Radiolabeling of the antibody conjugate

The ^213^Bi-labeling of the MX35 conjugate was performed and evaluated as previously described, [13] but with some modifications. A total of 0.1 mg of the antibody conjugate was added to the pH-adjusted ^213^Bi eluate, and the reaction was continued for 5 min. After quenching with 10 μl of 1.5 mg/mL 2-[Bis[2-bis(carboxymethyl)amino] ethyl]amino]acetic acid (DTPA), the labeled product was purified using a NAP-10 column (GE Healthcare) and eluted with PBS.

### Immunoreactivity of the ^213^Bi-labeled antibody conjugates

Prior to the in vivo experiments, the immunoreactivity of the radiolabeled MX35 was evaluated in vitro using suspensions of live OVCAR-3 cells as previously described [13]. Briefly, 10 ng of the labeled antibodies were added to duplicates of 0.5 mL of serially diluted cell suspensions (maximum 5 × 10^6^ cells/mL). The samples were incubated for 3 h at RT with vigorous agitation, centrifuged for 5 min, and washed twice with 1 mL of PBS. Following the last wash and centrifugation, the cell pellets were measured in a γ counter (Wallac, Finland) and compared with reference solutions of radiolabeled MX35. The fraction of bound activity over the total applied activity was calculated, as well as the immunoreactive fraction according to the Lindmo assay [21].

### Therapeutic efficacy and toxicity study

Therapeutic efficacy was investigated for two different activity levels of ^213^Bi-labeled MX35. Sixty mice were inoculated i.p. with 1 × 10^7^ OVCAR-3 cells. Two weeks after cell inoculation, 40 mice were injected i.p. with ^213^Bi-MX35: 20 were given 9 MBq/mL, equal to 30 μg of ^213^Bi-MX35 in 1 mL of PBS, and 20 were given 3 MBq/mL, equal to 10 μg of ^213^Bi-MX35 in 1 mL of PBS. A control group of mice received unlabeled MX35, 30 μg, in 1 mL PBS.

According to the ethical license, the mice were weighed prior to treatment and then every 7 to 10 days during treatment to monitor for any ascites discomfort. To monitor for acute myelotoxicity, white blood cell (WBC) counts and platelet counts in the blood from the tail vein of 5 mice from each group were analyzed at 6 and 14 days after treatment. The samples were analyzed in a microcell counter (Sysmex F-820; Toa Medical Electronics Co., Ltd., Kobe, Japan). The animals were sacrificed 8 weeks after therapy, and the abdominal cavity was opened to investigate any presence of ascites, microscopic, and/or macroscopic tumors. Microscopic tumor was defined as tumor sizes ranging from single cells to clusters of million cells not detected by the naked eye at autopsy, whereas macroscopic tumors are visible and detectable at autopsy, often more than 1 mm with or without ascites. To investigate occurrence of microscopic tumors, tissues from the abdominal wall, mesentery, and spleen were taken from all animals for paraffin sectioning and hematoxylin and eosin (H&E) staining. Additional samples were taken from suspected lesions at other locations. Between 10 and 30 sections at 50 μm distance were processed for microscopy. To support the H&E-stained slides in microscopic tumor identification, immunohistochemistry (IHC) for the MX35 antigen was also performed in representative cases. Both the animal dissection and the histological analysis were blinded, i.e., performed without any knowledge of the treatment received.

### Statistical analyses

Analyses were performed to evaluate possible statistical significance between the two treated groups and the control group. Calculations were performed for the two-sided alternative hypothesis using Fisher’s exact test.

### Dosimetric calculations

In the mouse model, the dose-limiting organ could possibly be the bone marrow. Previously presented biodistribution data for i.p. injected ^213^Bi-MX35 were used for bone marrow dosimetry [12, 13]. The time-integrated activity per unit mass, *Ã/m* (Bq s kg^−1^) was calculated from the area under the activity concentration curve for blood. The absorbed dose *D* was calculated as:

1 $$ D=\frac{\tilde{\mathrm{A}}}{m}\times \varDelta \times \varPhi $$

where Δ is the mean energy of the *α* particles per nuclear transformation for ^213^Bi (1.33 × 10^−12^ J), and *Φ* is the absorbed fraction of the *α* particles (assumed to be 1). A previously obtained bone marrow-to-blood ratio of 0.58 (for ^211^At-labeled MX35) [22] was then applied to estimate the resulting absorbed dose to the bone marrow of i.p. injected ^213^Bi-MX35.

The absorbed dose to the peritoneum was calculated as 50% of the equilibrium dose of the injected fluid. The antibody concentration in the i.p. fluid was assumed to be equal to the antibody concentration in the injected solution during the time of the ^213^Bi decay.

Biokinetic modeling and microtumor dosimetry was performed as previously described by Palm et al. [12]. The modeling was adjusted for mouse kinetics, for the specific activity achieved in the study and the activity concentrations used, i.e., 3 and 9 MBq/mL. The number of antigen per cell was set to 700 000. Microdosimetry was performed for single cells and clusters with radii 9, 30, and 50 μm.

## Results

### Antibody labeling and immunoreactivity

The radiochemical yield after the ^213^Bi-labeling was 53 ± 12% (mean value ± standard deviation; without correction for decay), and the radiochemical purity was 91 ± 6.0%. The cell-binding assay showed that the fraction of bound activity after 3 h was 0.83 for the highest concentration of cells in the cell suspensions. The immunoreactive fraction of the radiolabeled mAbs in vitro, i.e., the calculated fraction of conjugated antibodies able to bind to the cells in a situation of infinite antigen access, was plotted and calculated to be 0.91. The specific activity at the time of injection was on average 45.6 GBq/μmol, which corresponds approximately a radionuclide:antibody ratio of 1:3000.

### Therapeutic efficacy

**Fig. 1:**
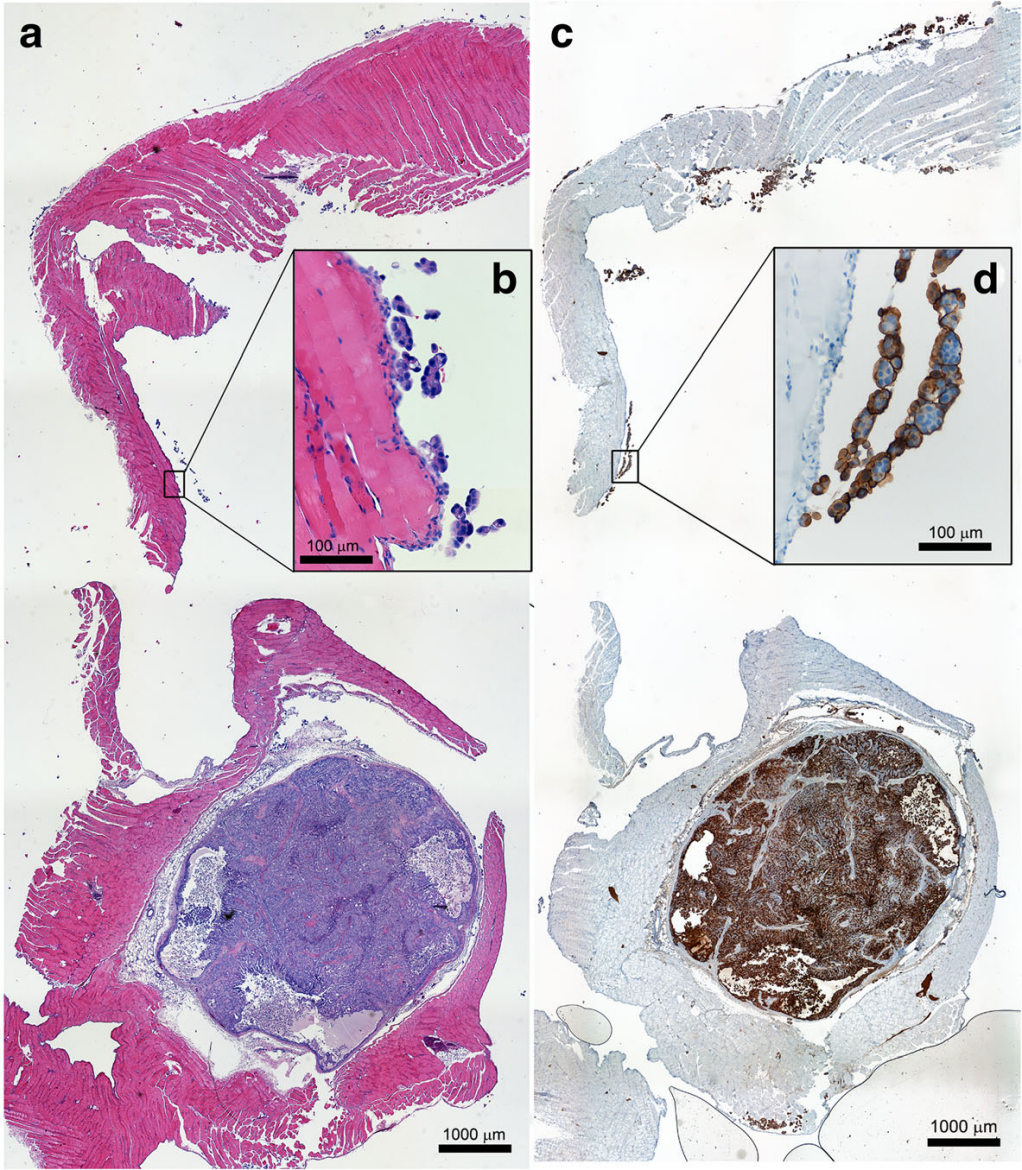
Tissue sections of the abdominal wall from a reference animal treated with unlabeled MX35 visualizing a macroscopic tumor (*bottom* of **a** and **c**) and microscopic tumors (**b** and **d**). **a** and **b** are stained with H&E while **c** and **d** show the dense distribution (in *brown*) of the MX35 antigen on tumor cells using IHC

The tumor-free fractions (TFFs) of the groups treated with 3 or 9 MBq/mL of ^213^Bi-labeled MX35 were 0.55 and 0.78, respectively, (Table 1). No animals in the groups receiving ^213^Bi-MX35 developed ascites. However, four animals in the control group receiving cold MX35 had obvious ascites production. The control group also had the largest fraction of animals with macroscopic tumor incidence. The overall TFF of the control group was 0.15 (Table 1). The samples taken for microscopy evaluation from the peritoneal cavity showed a wide range of tumor progression, from complete absence of visual tumor cells to large tumors. Examples of visualizations of macroscopic and microscopic tumors by H&E staining and IHC are shown in Fig. 1.

**Table 1:** Results after treatment with ^213^Bi-MX35 and unlabeled MX35

Group	Treatment	Activity concentration	Macroscopic tumor (no. of animals)	Microscopic tumor (no. of animals)	Ascites (no. of animals)	TFF
1	^213^Bi-MX35	3 MBq/mL	4/20	9/20	0	0.55
2	^213^Bi-MX35	9 MBq/mL	1/18^a^	4/18^a^	0	0.78
3	Ref. group (cold MX35)	–	7/20	17/20	4	0.15

*TFF* tumor-free fraction, i.e., fraction of the mouse groups with no macroscopic or microscopic tumors and no ascites

^a^Two animals were excluded from the mouse group receiving 9 MBq/mL; one was sacrificed earlier because of atypical mouse behavior (no tumor tissue could be detected), and one was excluded because of a sole subcutaneous tumor outside of the peritoneum which was suspected to be the result of cell leakage from the peritoneal cavity post inoculation

### Statistical analyses

The statistical analysis performed using Fisher’s exact test showed that the difference in TFF between the mouse group receiving 3 MBq/mL of ^213^Bi-MX35 and the control group and between the group receiving 9 MBq/mL of ^213^Bi-MX35, and the control group was statistically significant (*p* = 0.02) and (*p* = 0.0002) respectively. The difference between the group receiving 3 MBq of ^213^Bi-MX35 and the group receiving 9 MBq/mL of ^213^Bi-MX35 was, however, not statistically significant (*p* = 0.18).

### Toxicity

**Fig. 2:**
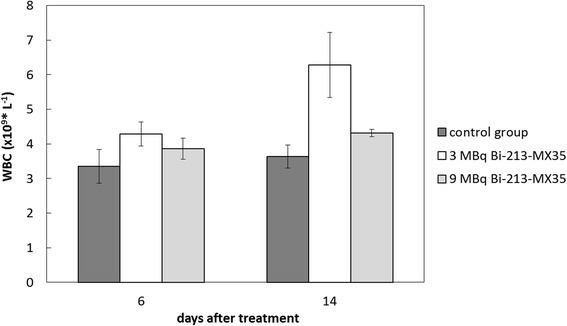
White blood cell (WBC) counts measured in the blood samples taken from the tail vein 6 and 14 days after treatment. Each group consisted of five animals. Means and standard error of the mean (SEM; *error bars*) are depicted

In the group treated with 3 MBq/mL of ^213^Bi-MX35, the WBC counts were on average 4.3 × 10^9^/L 6 days after treatment, as shown in Fig. 2. In the 9 MBq/mL group, the WBC counts were slightly lower with an average of 3.9 × 10^9^/L. However, the WBC counts of the control animals were 3.4 × 10^9^/L, i.e., lower than in both of the treated groups. Fourteen days after treatment, the WBC counts had increased by 22% on average in all animals. The increase in WBC counts was lowest in the control group (8%). Thus, no bone marrow toxicity could be observed from the WBC levels of the animals in any of the treated groups.

**Fig. 3:**
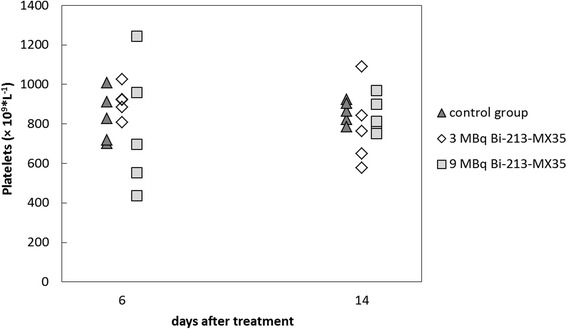
Platelet counts measured in the blood samples taken from the tail vein 6 and 14 days after treatment. Each group consisted of five animals

Neither could we demonstrate significant hematological toxicity from the platelet counts after any of the treatments, as shown in Fig. 3. The group receiving 9 MBq/mL of ^213^Bi-MX35 had the lowest platelet counts 6 days after treatment (mean value = 777 × 10^9^/L). However, the variation between the animals was large, and the mean value for the group receiving 9 MBq/mL was similar to the value of the control group 14 days after treatment.

### Dosimetric calculations

When using previous biodistribution data [13] to calculate the absorbed dose in the mice receiving 3 MBq/mL of ^213^Bi-MX35, the absorbed dose to blood was approximately 1.3 Gy, resulting in an estimated absorbed dose to the bone marrow of 0.8 Gy. Accordingly, the mice administered 9 MBq/mL of ^213^Bi-MX35 received an absorbed dose to blood of approximately 3.9 Gy, yielding an estimated dose to the bone marrow of 2.3 Gy. The absorbed dose to the kidneys was estimated to be 0.45 and 1.35 Gy for the 3 and 9 MBq/mL level, respectively. The injected activity concentrations of ^213^Bi-MX35 at 3 and 9 MBq/mL would result in absorbed doses to a human peritoneum of 7.9 and 23.6 Gy, respectively, according to the dosimetric calculations. The calculated dose to microtumors from both non-specific and specific, i.e., cell-bound, irradiation are shown in Table 2.

**Table 2:** Absorbed dose to tumors

	Radius (μm)	Specific absorbed dose (Gy)	Unspecific absorbed dose (Gy)	Total absorbed dose (Gy)
3 MBq/mL	9	3.1	7.8	10.9
	30	3.5	8.2	11.7
	50	4.1	7.2	11.3
9 MBq/mL	9	3.3	23.4	26.7
	30	3.8	24.6	28.4
	50	4.3	21.6	25.9

## Discussion

The current study was performed to evaluate the therapeutic efficacy of RIT with ^213^Bi in an ovarian cancer model at two activity concentration levels, by comparing a previously used administered activity concentration with an assumed maximum level tolerated by the mice. The mice were treated following 2–3 weeks post cell injection and were evaluated for tumor occurrence 8 weeks after treatment. The end point, tumor-free fraction at 8 weeks post injection was chosen to avoid debilitating symptoms such as severe ascites before 8 weeks and to be able to compare with previously published results using this endpoint. While the peritoneum might be the critical normal organ in humans, the bone marrow sets the limit for administered activity concentration in mice. This difference is due to the higher transport rate from the peritoneal fluid to the systemic circulation in mice. In humans, this rate is slow compared mice and to the half-life of ^213^Bi. In our previous studies, we observed that the mice could tolerate 2 Gy to the bone marrow from *α* radiation, but that 3 Gy could lead to lethal myelotoxicity. For the highest administered activity group in the current study, with an estimated absorbed dose to the bone marrow of 2.3 Gy, the WBC counts did not appear affected to any significant extent. Another potential organ at risk with ^213^Bi is the kidneys. In the current study, the absorbed dose to the kidneys was 0.45 and 1.35 Gy, which according to previous studies is well below the tolerance dose [23, 24].

The current study showed that both 3 and 9 MBq/mL of ^213^Bi-MX35 had a significant effect on microscopic tumors in the mouse model. The TFF found for 3 MBq/mL in the current study (0.55) was in good agreement with results from a previous study (0.60) aimed at comparing ^213^Bi with ^211^At [13]. The current study also showed that the TFF was further improved (0.78) by increasing the administered activity concentration to 9 MBq/mL, but this difference was not statistically significant.

Calculated dosimetry indicates that the microtumor irradiation from the ^213^Bi-mAbs with the specific activity obtained in this study will have its main origin from surrounding, non-targeted ^213^Bi-mAbs in the i.p. fluid. This non-specific irradiation does not depend on tumor cell antigen expression or on the specific activity of the labeled compound. In the current study, a specific activity high enough to result in eradicative doses from cell-bound ^213^Bi-mAbs was not achieved. The specific irradiation is only slightly higher for the 9 MBq/ml activity concentration as antigen saturation occurs within a few minutes after injection for both activity concentrations Thus, the therapeutic effect is explained mainly by the non-specific *α* irradiation that linearly increases with activity concentration. However, the actual size of the tumors at the time of the treatment in this tumor model is not known. An indication of the size was obtained from histological samples after treatment, and it describes a large individual variation with sizes between barely visible to large tumor clusters. The therapeutic effect may therefore in this study to some extent also be related to the variation of tumor burden among the mice at the time of treatment [2]. To increase the absorbed dose to tumor clusters larger than the range of the alpha particles, a fractionated treatment schedule could possibly improve the therapeutic outcome.

The half-life of ^213^Bi results in a low bone marrow dose in humans, allowing administration of high activities. The resulting non-specific irradiation is significant and can be of clinical advantage in e.g., humans where the targeted antigen expression might be low or lacking in a subpopulation of cells. Likewise, in the case of low-specific activity, i.e., a low nuclide to antibody ratio, of the ^213^Bi-mAb product, a high administered activity concentration could still lead to non-specific irradiation that could eradicate microtumors. However, achieving a high-specific activity product would increase the tumor dose, increasing the probability of cure.

We have previously shown that absorbed doses up to 30–50 Gy [25] results in a very limited effect on the peritoneum in mice. However, translation of the data retrieved from the current and previous animal studies to a human situation is difficult to make and can at this stage only be given as an approximation. Although the peritoneal sensitivity to *α* radiation needs to be further studied before a tolerance dose can be estimated, it is likely that the highest administered activity concentration in the current study, which translates to 9 GBq in 1 L and 24 Gy to a human peritoneum, would be within a safe interval in a clinical situation. This is due to the distance between the peritoneal lining and the radiosensitive intestinal crypts being greater than the range of the emitted *α* particles, thereby eliminating the risk of intestinal toxicity. Following administration of 9 GBq/L, the corresponding estimated absorbed dose to the bone marrow in a patient will be very low, about 0.02 Gy [11]. The absorbed dose to other risk organs in the patient, e.g., the kidney will also be very low. This is due do the half-life of ^213^Bi and the slow clearance from the peritoneal cavity, i.e., there will be a very limited systemic distribution/irradiation following i.p. administration of ^213^Bi labeled antibodies.

The current study shows some promise for i.p. [^213^Bi]-RIT treatment of microscopic ovarian cancer. However, obstacles remain including, e.g., availability of the radionuclide in order to achieve activity levels suitable for scale up to clinical treatment and obtaining the required specific activity of the ^213^Bi-mAb.

## Conclusions

Tumor growth after i.p. treatment with ^213^Bi-MX35 was reduced compared to treatment with unlabeled MX35. Compared with 3 MBq/mL of ^213^Bi-MX35, treatment with 9 MBq/mL of ^213^Bi-MX35 resulted in higher non-specific irradiation and a tendency of higher TFF. No signs of toxicity were observed in the treated animals. Although the unspecific irradiation contribute to a high absorbed dose to tumor cells in i.p. therapy, higher specific activity of the ^213^Bi-mAb is desirable to increase the specific irradiation of the tumor cells and ultimately improve the therapeutic outcome.

## Abbreviations

H&E: Hematoxylin and eosin
i.p.: Intraperitoneal
IHC: Immunohistochemistry
mAbs: Monoclonal antibodies
PBS: Phosphate buffered saline
RIT: Radioimmunotherapy
RT: Room temperature
TFF: Tumor-free fraction
WBC: White blood cell
α-RIT: Alpha-particle radioimmunotherapy
